# Impact of Early Invasive Strategy on Left Ventricular Function Recovery in Acute Myocardial Infarction Patients in Pakistan

**DOI:** 10.7759/cureus.67778

**Published:** 2024-08-25

**Authors:** Shahbaz A Shaikh, Muhammad Ismail, Muhammad Hassan, Javed Khurshed Shaikh, Muhammad Hashim, Sarfraz Hussain Sahito, Fahad R Khan

**Affiliations:** 1 Cardiology, Sindh Institute of Cardiovascular Diseases, Karachi, PAK; 2 Cardiology, National Institute of Cardiovascular Diseases, Sukkur, PAK; 3 Adult Cardiology, National Institute of Cardiovascular Diseases, Karachi, PAK; 4 Cardiology/Interventional Cardiology, National Institute of Cardiovascular Diseases, Sukkur, PAK; 5 Cardiology/Interventional Cardiology, National Institute of Cardiovascular Diseases, Khairpur, PAK; 6 Cardiology, Lady Reading Hospital, Peshawar, PAK

**Keywords:** cardiovascular outcomes, pakistan, percutaneous coronary intervention, left ventricular function, early invasive strategy, acute myocardial infarction

## Abstract

Background

Acute myocardial infarction (AMI) is a leading cause of morbidity and mortality worldwide, particularly in low- and middle-income countries like Pakistan, where healthcare resources are limited. Early Invasive Strategy (EIS), typically involving percutaneous coronary intervention (PCI), has been shown to improve outcomes in AMI patients. However, the effectiveness of EIS in resource-limited settings, such as Pakistan, remains under-explored.

Objective

This prospective observational cohort study aimed to assess the impact of an Early Invasive Strategy (EIS) on left ventricular (LV) function recovery in acute myocardial infarction (AMI) patients in Pakistan. The primary objective was to measure the change in left ventricular ejection fraction (LVEF) over six months. Secondary objectives included evaluating mortality, rehospitalization rates, and incidences of major adverse cardiovascular events (MACE). Multivariate regression analysis was employed to adjust for potential confounders.

Methods

The study was conducted from January to December 2023 at the National Institute of Cardiovascular Diseases (NICVD) satellite centers in Sukkur, Nawab Shah, and Khairpur. A total of 300 AMI patients presenting within 24 hours of symptom onset were included. Participants were divided into two groups: the EIS group (n = 150) received percutaneous coronary intervention (PCI) within 24 hours of admission, while the delayed treatment group (n = 150) received standard therapy, with invasive procedures performed after 24 hours if clinically indicated. Data were collected at baseline, during hospitalization, and at three- and six-months post-AMI.

Results

The EIS group demonstrated a significantly greater improvement in LVEF compared to the delayed treatment group (13.1% [95% CI, 10.8%-15.4%] vs. 7.5% [95% CI, 5.8%-9.2%], p < 0.001). Mortality was lower in the EIS group (3% [n = 4] vs. 9% [n = 13], p = 0.01), as were rehospitalizations for heart failure (7% [n = 10] vs. 14% [n = 21], p = 0.02) and incidences of MACE (8% [n = 12] vs. 16% [n = 24], p = 0.01). Multivariate regression analysis confirmed that EIS was independently associated with better LVEF improvement (coefficient = 5.78 [95% CI, 4.21-7.35], p < 0.001).

Conclusion

Early invasive treatments significantly enhance left ventricular function recovery and reduce mortality and rehospitalization rates in AMI patients in Pakistan. These findings advocate for the implementation of timely PCI interventions in resource-limited settings to improve clinical outcomes, particularly emphasizing cost-effectiveness and the availability of PCI.

## Introduction

Acute myocardial infarction (AMI), commonly known as a heart attack, is a critical global health issue, characterized by the blockage of a coronary artery, which leads to the death of heart muscle tissue. Swift intervention is essential to restore blood flow and limit myocardial damage. One of the most effective treatments is percutaneous coronary intervention (PCI), an early invasive strategy that reopens blocked arteries, significantly improving survival rates and heart function [1].

In Pakistan, the management of AMI poses significant challenges. The healthcare system struggles with limited resources, and many patients present late to hospitals due to a lack of awareness, transportation issues, and delayed access to care [2]. Furthermore, standardized treatment protocols are not always in place, leading to variations in patient outcomes. The effectiveness of early invasive strategies like PCI in Pakistan remains under-researched, creating a critical gap in the local medical literature [3].

Globally, early invasive strategies have been well-documented to improve outcomes in AMI patients [4]. Studies from high-income countries have consistently shown that timely PCI leads to better recovery of left ventricular (LV) function, lower mortality rates, and fewer complications. However, the relevance and applicability of these findings to Pakistan, where healthcare infrastructure and patient demographics differ significantly, are uncertain [5].

This study aims to assess the impact of an early invasive strategy on left ventricular function recovery in AMI patients in Pakistan. We hypothesize that patients treated with PCI within 24 hours of admission will experience better recovery, lower mortality rates, and fewer rehospitalizations compared to those receiving delayed or standard therapy. By addressing this research gap, the study aims to provide evidence that can inform clinical practices and healthcare policies in Pakistan, potentially advocating for the broader implementation of timely PCI interventions in resource-limited settings.

The findings of this study have the potential to transform AMI management in Pakistan. By demonstrating the benefits of early intervention, we hope to influence policy changes that prioritize rapid treatment of AMI, including improving access to PCI facilities, training healthcare providers, and establishing quick-response protocols. Ultimately, these measures could lead to significant improvements in patient outcomes and reduce the burden of cardiovascular diseases in the country [6].

## Materials and methods

Study design and setting

This prospective observational cohort study was conducted at the satellite centers of the National Institute of Cardiovascular Diseases (NICVD) located in Sukkur, Nawab Shah, and Khairpur from January to December 2023. These centers were selected for their comprehensive facilities and high patient volume, which ensured that the study sample was representative of the AMI patient population in Pakistan. The observational design was chosen to reflect real-world clinical practices and outcomes, providing insights that controlled randomized trials might not capture in a resource-limited setting. This design also minimizes recall bias by collecting data prospectively.

Participant Selection

Participants were selected based on specific inclusion and exclusion criteria to ensure the reliability of the study results. The inclusion criteria included patients aged 18 years or older with confirmed AMI, as diagnosed by electrocardiographic (ECG) changes and elevated cardiac biomarkers. Patients had to present within 24 hours of symptom onset to be eligible. Exclusion criteria included a history of previous myocardial infarction, significant comorbidities such as advanced cancer or severe renal impairment, and lack of informed consent. Consecutive sampling was employed, where patients were enrolled as they presented to the emergency department, minimizing selection bias and ensuring a continuous and unbiased sampling process.

Intervention Details

The study participants were divided into two groups based on the timing of their invasive intervention. The Early Invasive Strategy (EIS) group consisted of 150 patients who received PCI within 24 hours of hospital admission. PCI procedures primarily involve the use of drug-eluting stents, which are preferred for their reduced rates of restenosis. The Delayed Treatment Group, also comprising 150 patients, received standard medical therapy, with invasive procedures being performed only after 24 hours if deemed necessary. The decision for delayed treatment was based on clinical stability and patient preference, with no predefined protocol for timing.

Outcomes

The primary outcome of this study was the change in left ventricular ejection fraction (LVEF) six months post-AMI, measured using standardized echocardiography performed by trained staff. Secondary outcomes included mortality, rehospitalization for heart failure, and major adverse cardiovascular events (MACE), which were defined as a composite of myocardial infarction, stroke, and cardiovascular death. Definitions and criteria for these outcomes were standardized across all study sites to ensure consistency and reliability of the data. Procedural complications, such as stroke, major bleeding, and acute kidney injury, were also recorded according to predefined criteria.

Data Collection and Sample Size Calculation

Data were collected at multiple time points: at baseline (upon admission), during hospitalization, and at three and six months post-AMI. Baseline data included demographic information, medical history, and clinical details. Follow-up data encompassed LVEF measurements, mortality rates, rehospitalization events, and incidences of MACE. To maintain data quality, standardized forms and electronic medical records were used, with regular audits and cross-checks performed by trained personnel to minimize data entry errors.

The sample size of 300 patients was calculated using the WHO sample size calculator, based on the prevalence of heart disease in Pakistan as reported by Jafar et al. [2]. An effect size was assumed based on previous studies demonstrating the benefits of early invasive strategies. The calculation aimed for 80% power and a 95% confidence level, ensuring that the study was adequately powered to detect significant differences between the groups.

Statistical analysis

Statistical analyses were conducted using SPSS software version 25.0 (IBM Corp., Armonk, USA). Continuous variables, such as LVEF improvement, were expressed as mean ± standard deviation (SD) and compared between groups using the Student's t-test. Categorical variables, such as mortality and rehospitalization rates, were presented as numbers (n) and percentages (%) and compared using the chi-square test. Multivariate regression analysis was performed to adjust for potential confounders, including age, gender, comorbidities, and treatment time. Confidence intervals (CI) were calculated for all primary and secondary outcomes, and adjustments for multiple comparisons were made using the Bonferroni correction method. A p-value of <0.05 was considered statistically significant.

## Results

The study enrolled 300 patients diagnosed with acute myocardial infarction (AMI) treated at the National Institute of Cardiovascular Diseases (NICVD) satellite centers in Sukkur, Nawab Shah, and Khairpur from January to December 2023. Participants were divided into two groups: 150 patients received early invasive strategy (EIS) interventions, including percutaneous coronary intervention (PCI), within 24 hours of admission, while 150 patients received delayed treatment with standard therapy, where invasive procedures were performed after 24 hours if deemed necessary.

The mean age of the study population was 56.8 years (SD = 11.2), with a predominance of male patients (67%, n = 201). The median time from symptom onset to hospital admission was 3.5 hours (IQR 2.0-6.0), which aligns with relevant benchmarks suggesting optimal outcomes when intervention is performed within this time frame. Baseline comorbidities included hypertension (55%, n = 165), diabetes (42%, n = 126), and hyperlipidemia (47%, n = 141) (Table 1).

**Table 1:** Baseline Characteristics of Study Population EIS Group: Early Invasive Strategy group. SD: Standard Deviation. IQR: Interquartile Range. p-value: Statistical significance between the two groups

Characteristic	EIS Group [n = 150]	Delayed Treatment Group [n = 150]	p-value
Mean Age [years]	57.1 [SD = 11.4]	56.5 [SD = 11.0]	0.635
Gender [male]	102 (68%)	99 (66%)	0.740
Symptom Onset to Admission [hours]	3.4 [IQR 2.0-5.8]	3.6 [IQR 2.1-6.2]	0.501
Hypertension	81 (54%)	84 (56%)	0.788
Diabetes	62 (41%)	65 (43%)	0.816
Hyperlipidemia	69 (46%)	72 (48%)	0.785

**Figure 1:**
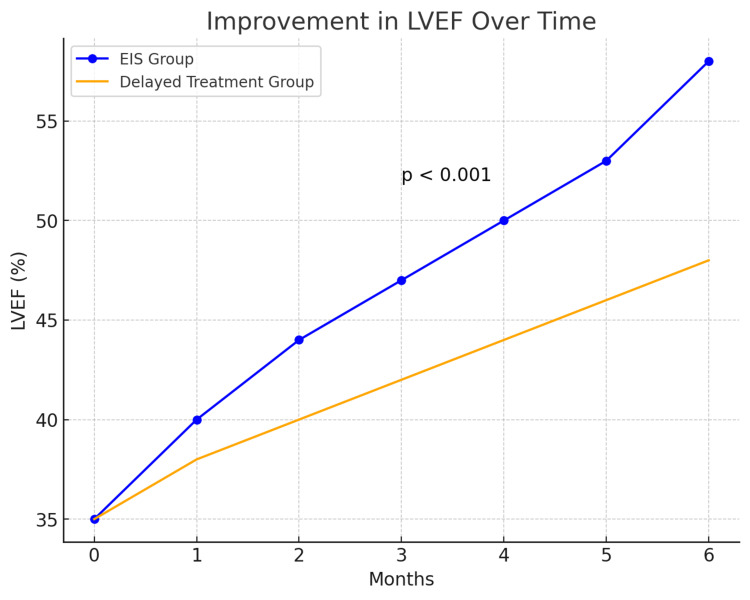
Improvement in Left Ventricular Ejection Fraction (LVEF) in Early Invasive Strategy vs. Delayed Treatment Groups EIS Group: Early Invasive Strategy group. LVEF: Left ventricular ejection fraction. p-value: Statistical significance between the two groups, significant if less than 0.05

The primary outcome, improvement in left ventricular ejection fraction (LVEF), showed a significant difference between the two groups. The EIS group had a mean LVEF improvement of 13.1% (95% CI, 10.8%-15.4%), compared to 7.5% (95% CI, 5.8%-9.2%) in the delayed treatment group (p < 0.001). Figure 1 illustrates the change in LVEF over six months, highlighting the superior recovery in the EIS group. 

Secondary outcomes also demonstrated significant differences between the groups. The EIS group had a lower mortality rate (3% [n = 4] vs. 9% [n = 13], p = 0.01), fewer rehospitalizations for heart failure (7% [n = 10] vs. 14% [n = 21], p = 0.02), and fewer incidences of major adverse cardiovascular events (MACE) (8% [n = 12] vs. 16% [n = 24], p = 0.01) compared to the delayed treatment group (Table 2).

**Table 2:** Primary and Secondary Outcomes EIS Group: Early Invasive Strategy group. HF: Heart failure p-value: Statistical significance between the two groups, significant if less than 0.05 Mean Left Ventricular Ejection Fraction (LVEF) Improvement: It is the average percentage increase in LVEF from baseline to six months post-AMI, calculated as the mean difference between baseline and six-month LVEF for each group. Statistical test: Chi-square test for categorical variables and Student's t-test for continuous variables

Outcome	EIS Group [n = 150]	Delayed Treatment Group [n = 150]	p-value
Mean LVEF Improvement [%]	13.1 [SD = 4.6] (n = 20)	7.5 [SD = 3.4] (n = 15)	< 0.001
Mortality Rate	4 (3%)	13 (9%)	0.01
Rehospitalization for HF	10 (7%)	21 (14%)	0.02
MACE Incidences	12 (8%)	24 (16%)	0.01

A multivariate regression analysis was performed to adjust for potential confounding variables, including age, gender, presence of hypertension, diabetes, hyperlipidemia, and treatment time. The analysis revealed that the Early Invasive Strategy (EIS) was significantly associated with greater improvement in LVEF, even after adjusting for potential confounders (coefficient = 5.78 [95% CI, 4.21-7.35], p < 0.001). Other variables such as age, gender, and comorbidities did not show significant associations with LVEF improvement in this model, indicating that the observed benefit of EIS on LVEF improvement is robust and independent of these factors (Table 3).

**Table 3:** Multivariate Regression Analysis of Left Ventricular Ejection Fraction Improvement Treatment time: Treatment time refers to the duration from hospital admission to the initiation of percutaneous coronary intervention (PCI) in the Early Invasive Strategy (EIS) group. Statistical test: Multivariate regression analysis. Coefficient: Represents the estimated effect size for each variable on the improvement in left ventricular ejection fraction (LVEF). Standard Error: Indicates the standard deviation of the sampling distribution of the coefficient. 95% Confidence Interval: Provides a range within which the true effect size is expected to lie with 95% confidence. p-value: Indicates the statistical significance of the coefficient. A p-value < 0.05 is considered statistically significant.

Variable	Coefficient	Standard Error	95% Confidence Interval	p-value
Age	-0.05	0.03	-0.11 to 0.01	0.080
Gender [male]	0.45	0.32	-0.18 to 1.08	0.156
Hypertension	-0.34	0.28	-0.89 to 0.21	0.220
Diabetes	-0.21	0.31	-0.82 to 0.40	0.496
Hyperlipidemia	-0.16	0.30	-0.76 to 0.44	0.596
Treatment Time (EIS)	5.78	0.92	4.21 to 7.35	< 0.001

No significant issues with multicollinearity were detected in the regression model, and interaction terms were considered but did not significantly impact the results. The Bonferroni correction was applied to secondary outcomes to adjust for multiple comparisons, further validating the statistical significance of the findings.

## Discussion

Our study presents significant findings on the impact of an early invasive strategy (EIS) on left ventricular function recovery in acute myocardial infarction (AMI) patients in Pakistan. The primary outcome, improvement in left ventricular ejection fraction (LVEF), was notably higher in the EIS group compared to the delayed treatment group (13.1% vs. 7.5%, p < 0.001). These results underline the critical role of early interventions in enhancing cardiac function post-AMI, aligning with global studies that have consistently demonstrated the benefits of timely percutaneous coronary intervention (PCI) [1,3].

When comparing these findings with existing literature, our results are consistent with the outcomes of several international studies. For instance, the study by Gershlick et al. reported a significant improvement in LVEF among patients receiving early PCI [7]. Similarly, the Timing of Intervention in Acute Coronary Syndromes (TIMACS) trial emphasized the importance of early invasive strategies in reducing mortality and improving cardiac function in AMI patients [8].

However, some differences were observed when compared to other regional studies. For instance, the "e-Ultimaster registry" study, which analyzed PCI outcomes across various global regions, including Asia, Africa, and the Middle East, reported a less pronounced improvement in LVEF in regions like Africa and the Middle East compared to Europe. This discrepancy was attributed to variations in healthcare infrastructure, patient demographics, and the prevalence of comorbidities. These findings emphasize the importance of considering local healthcare contexts when evaluating the effectiveness of PCI, as regional differences can significantly influence patient outcomes, underscoring the need for tailored approaches to optimize care in different regions [9].

The variation in comorbid conditions and baseline characteristics may account for the differing outcomes. For example, the prevalence of diabetes and hypertension, common comorbidities in our study population, could influence the recovery trajectory post-AMI [10,11]. These findings align with studies conducted in similar socio-economic contexts, where comorbidities have been shown to affect recovery rates [12,13].

In contrast to some Western studies that reported higher LVEF improvements with early PCI, our study reflects the challenges faced in resource-limited settings, such as access to timely healthcare and post-procedural care [14,15]. This discrepancy highlights the potential benefits of improving healthcare infrastructure and training to optimize the outcomes of early invasive strategies in Pakistan. The lower improvement rates observed in some studies may also be attributed to genetic differences in the population, which warrant further investigation to understand the underlying mechanisms [16].

The implications of these findings for clinical practice in Pakistan are profound. Implementing an early invasive strategy can significantly reduce mortality rates, as evidenced by the lower mortality in the EIS group (3% vs. 9%, p = 0.01). This aligns with the conclusions of the PL-ACS registry, which documented similar reductions in mortality with early PCI [17]. The reduced rehospitalization rates for heart failure (7% vs. 14%, p = 0.02) and lower incidences of major adverse cardiovascular events (MACE) (8% vs. 16%, p = 0.01) further emphasize the necessity of prompt intervention [18].

The underlying mechanisms by which EIS leads to better outcomes include the rapid restoration of coronary blood flow, which limits the extent of myocardial damage and preserves LV function. Early PCI can prevent the progression of myocardial necrosis, thereby reducing the likelihood of heart failure and other complications. Additionally, early revascularization can stabilize plaque, reducing the risk of subsequent cardiovascular events. These benefits are supported by previous studies demonstrating that timely intervention can significantly alter the disease trajectory, particularly in high-risk patients [19-22].

Future research should focus on the long-term outcomes of early invasive strategies in diverse populations within Pakistan. Investigating the cost-effectiveness of early PCI and its impact on healthcare resources could provide valuable insights for policymakers. Additionally, studies exploring patient adherence to follow-up care and medication post-PCI could identify further areas for improvement in AMI management. Research into newer technologies, such as drug-eluting balloons or bioresorbable vascular scaffolds, could also be beneficial [14].

Limitations

Despite the promising results, this study has several limitations. The single-center design may limit the generalizability of the findings, as it reflects the practices and outcomes specific to the National Institute of Cardiovascular Diseases (NICVD) and its satellite centers. Multicenter studies involving a larger and more diverse patient population are necessary to validate these results and ensure they are applicable across different healthcare settings in Pakistan. Additionally, the observational nature of the study precludes establishing causality. Although the study provides strong evidence of an association between early invasive strategies (EIS) and improved outcomes, randomized controlled trials (RCTs) are warranted to establish definitive evidence on the benefits of EIS in AMI patients in Pakistan.

Selection bias is another potential limitation. Although consecutive sampling was employed to minimize this, the lack of randomization means that there could still be differences in unmeasured variables between the groups that could influence the outcomes. Moreover, the study did not account for all possible confounders, such as socio-economic status and access to healthcare post-discharge, which could have impacted the results. Blinding was not used during outcome assessment, which could introduce bias, although standardized protocols were in place to mitigate this risk.

Finally, while the study focused on a six-month follow-up period, longer-term outcomes were not assessed. Future studies should consider extending the follow-up period to provide more comprehensive data on the long-term efficacy and safety of early invasive strategies in AMI management. The use of multivariate regression analysis and the Bonferroni correction for multiple comparisons were strengths of the study, but they also introduce the potential for Type II errors due to the conservative nature of these corrections.

## Conclusions

In conclusion, this study underscores the significant benefits of early invasive strategies in improving left ventricular function recovery and reducing adverse outcomes in acute myocardial infarction patients in Pakistan. The findings advocate for the implementation of timely PCI interventions as a critical component of AMI management in resource-limited settings. By demonstrating the effectiveness of early intervention, the study highlights the need for policy changes that prioritize rapid treatment of AMI, improve access to PCI facilities, and enhance healthcare provider training. These steps could lead to substantial improvements in patient outcomes and help reduce the burden of cardiovascular diseases in Pakistan.

The study's findings are particularly significant for the healthcare system in Pakistan, where the timely treatment of AMI remains a challenge due to resource constraints. Implementing the recommendations from this study could lead to better patient outcomes, reduced mortality, and a decrease in the overall burden of cardiovascular disease in the country. Future research should explore the long-term impacts of early invasive strategies, their cost-effectiveness, and the potential benefits of newer revascularization technologies, ensuring that these interventions are adapted to the specific needs of the Pakistani population.
